# Depolarization potentiates TRAIL-induced apoptosis in human melanoma cells: Role for ATP-sensitive K^+^ channels and endoplasmic reticulum stress

**DOI:** 10.3892/ijo.2012.1483

**Published:** 2012-05-17

**Authors:** YOSHIHIRO SUZUKI, TOSHIO INOUE, MAYUMI MURAI, MIKI SUZUKI-KARASAKI, TOYOKO OCHIAI, CHISEI RA

**Affiliations:** 1 Division of Molecular Cell Immunology and Allergology, Nihon University Graduate School of Medical Science, Tokyo;; 2 Department of Dermatology, Nihon University Surugadai Hospital, Tokyo, Japan

**Keywords:** TRAIL, depolarization, melanoma, K_ATP_, apoptosis, endoplasmic reticulum stress

## Abstract

Tumor necrosis factor-related apoptosis-inducing ligand (TRAIL) is promising for cancer treatment owing to its selective cytotoxicity against malignant cells. However, some cancer cell types, including malignant melanoma cells, are resistant to TRAIL-induced apoptosis. Therefore, drugs that can amplify TRAIL cytotoxicity are urgently required. Depolarization of the plasma membrane potential is associated with apoptosis induced by a variety of death-inducing agents but its role in apoptosis remains a matter of debate. We found that TRAIL treatment resulted in robust depolarization in human melanoma cells with a considerable lag (2–4 h). Moreover, membrane-depolarizing agents, including K^+^ and ATP-sensitive K^+^ (K_ATP_) channel inhibitors glibenclamide and U37883A enhanced TRAIL-induced apoptosis. On the contrary, inhibitors of calcium- and voltage-dependent K^+^ channels and mitochondrial K_ATP_ channels had no such effects. Melanocytes were insensitive to TRAIL-induced depolarization and apoptosis as well as to the sensitization by membrane-depolarizing agents despite their substantial surface expression of death receptors. TRAIL induced robust activation of X-box-binding protein-1 and caspase-12, both of which were enhanced by the K^+^ and K_ATP_ channel inhibitors, but not by other K^+^ channel inhibitors. Finally, caspase-12-selective inhibitor completely abolished the amplification of apoptosis. These findings suggest that depolarization promotes endoplasmic reticulum stress-mediated death pathway, thereby amplifying TRAIL cytotoxicity. Thus, membrane-depolarizing agents such as K_ATP_ channel inhibitors may have therapeutic potential in the treatment of TRAIL-resistant cancer cells without impairing tumor-selectivity.

## Introduction

Tumor necrosis factor-related apoptosis-inducing ligand (TRAIL) is a member of the tumor necrosis factor cytokine family and has a homotrimeric structure. TRAIL has been shown to induce apoptosis in cancer cells with minimal cytotoxicity toward non-transformed cells. TRAIL exerts its pro-apoptotic effect by binding to two death domain-containing receptors, TRAIL receptor 1 (TRAIL-R1)/death receptor (DR) 4 and TRAIL-R2/DR5 (1). Binding of TRAIL to TRAIL-R1/2 expressed on the cell surface initiates the extrinsic apoptotic pathway. TRAIL binding to these DRs induces their oligomerization and conformational changes in the death domains, resulting in receptor activation and the formation of a death-inducing signaling complex. This complex formation allows the binding of an adaptor molecule, Fas-associated protein with death domain, via death domain interactions. The adaptor molecule also contains a death effector domain that binds to caspase-8, resulting in their oligomerization and autoactivation (2). Activated caspase-8 in turn activates the effector caspase-3/6/7 that executes the apoptotic process. In some cell types (type I), the extrinsic pathway is sufficient to commit the cell to apoptosis, while in other cell types (type II), activation of caspase-8 is low. In the latter cells, amplification by the intrinsic mitochondrial pathway is necessary to evoke substantial apoptosis (3). This amplification is triggered by activated caspase-8. Activated caspase-8 can cleave and activate the pro-apoptotic Bcl-2-family molecule Bid. In turn, truncated Bid activates the other Bcl-2-family molecules, Bax and Bak, which results in their oligomerization and the formation of megachannels in the outer mitochondrial membrane. The release of cytochrome c through the Bax/Bak megachannels into the cytosol induces the assembly of the apoptosome, representing the activation-platform for caspase-9 (4,5). Caspase-9 also promotes the activation of the effector caspase-3/6/7, thereby providing a positive loop of caspase activation (3).

TRAIL is a promising drug in cancer treatment due to its selective cytotoxicity toward malignant cells. However, a growing body of evidence suggests that some cancer cell types, including malignant melanoma cells, are resistant to TRAIL-induced apoptosis despite their expression of death-inducing TRAIL-Rs on the cell surface (6). Moreover, TRAIL-responsive tumors acquire a resistant phenotype that renders TRAIL therapy ineffective. Although many possible mechanisms, including intracellular mechanisms, have been identified, the cause of the TRAIL-resistance remains under intense scrutiny, as these mechanisms contribute to TRAIL-resistance to varying extents in different tumor cells. In any case, overcoming the TRAIL-resistance of cancer cells is necessary for effective TRAIL therapy, and small molecular compounds that can amplify TRAIL-induced apoptosis are urgently required.

Impairment of the intracellular ion homeostasis leads to depolarization of the plasma membrane potential (7–9). Depolarization has been shown to be an early event in the apoptosis induced by divergent agents, including Fas (10), rote-none (11) and arsenic trioxide (12) and is considered to play an important pro-apoptotic role. By contrast, depolarization has also been shown to exhibit anti-apoptotic effects. Various membrane-depolarizing agents, including ouabain, tetraethylammonium (TEA) and veratridine, protect Purkinje cells against apoptosis (13). In addition, K^+^ loading and several K^+^ channel inhibitors protect various human tumor cells against staurosporine-induced apoptosis (14). These observations suggest that depolarization can act in both a pro-and anti-apoptotic manner depending on the cell types and the apoptotic stimuli involved. However, the cellular and molecular mechanisms underlying these dual functions remains unclear.

The role of depolarization in TRAIL-induced apoptosis is poorly documented. In the present study, we investigated whether TRAIL caused depolarization and, if so, explored its role in TRAIL-induced apoptosis. Results revealed that TRAIL induced robust depolarization in melanoma cells, but not in normal melanocytes. Moreover, membrane-depolarizing agents such as K^+^ and K_ATP_ channel inhibitors sensitized melanoma cells, but not melanocytes, to TRAIL-induced apoptosis by promoting endoplasmic reticulum (ER) stress-mediated death pathway, including caspase-12.

## Materials and methods

### Reagents

Soluble recombinant human TRAIL and K^+^ channel inhibitors, glibenclamide (GLB), U37883A (U37), tetraethylammonium (TEA), 5-hydroxydecanoate (5-HD), α-dendrotoxin (DTX), charybdotoxin (CTX), *bis*-oxonol and DiBAC_4_(3) were obtained from Enzo Life Sciences (San Diego, CA, USA). In the present study, TRAIL was used at concentrations of 6.3–100 ng/ml and K^+^ channel inhibitors were used at 100 *μ*M except for DTX and CTX, both of which were used at 100 nM. Thapsigargin (Tg) was obtained from Sigma-Aldrich (St. Louis, MO, USA). The cell-permeable general caspase inhibitor z-VAD-fmk (VAD) and caspase-3/7-specific inhibitor z-DEVD-fmk (DEVD) were obtained from Calbiochem (La Jolla, CA, USA). The caspase-12-specific inhibitor z-ATAD-fmk (ATAD) was purchased from BioVision (Mountain View, CA, USA). The reagents were dissolved in dimethylsulfoxide and diluted with HBSS to a final concentration of <0.1% before use and used at 10 or 30 *μ*M. Polyclonal antibodies against X-box-binding protein-1 (XBP-1) and glucose-related protein 78 (GRP78) were obtained from Santa Cruz Biotechnology (Santa Cruz, CA, USA). All other chemicals were of analytical grade.

### Cells

Human melanoma cell lines were obtained from RIKEN Bioresource Center Cell Bank (Tsukuba, Japan), Health Science Research Resource Bank (Osaka, Japan) and American Type Culture Collection (Manassas, VA, USA), and cultured in high glucose-containing DMEM (Sigma-Aldrich) supplemented with 10% FBS in a 5% CO_2_-containing atmosphere. The cells were harvested by incubation in HBSS containing 1 mM EDTA and 0.25% trypsin for 5 min at 37°C. Normal human epidermal melanocytes were obtained from Cascade Biologica (Portland, OR, USA) and cultured according to the manufacturer’s instructions.

### Measurement of depolarization

Depolarization was measured by flow cytometry using *bis*-oxonol or DiBAC_4_(3), an anionic dye that shows an increase in fluorescence intensity upon membrane depolarization, as previously described (15). Cells (4×10^5^ cells/500 *μ*l) suspended in HBSS were incubated with 100 nM dye for 15 min at 37°C, and then incubated with the agents to be tested for 2–4 h at 37°C in a 5% CO_2_-containing atmosphere. Subsequently, 1×10^4^ cells were counted for their fluorescence using the FL-2 channel of a FACSCalibur (BD Bioscience, San Jose, CA, USA) and analyzed using the CellQuest software (BD Bioscience). For analysis of the depolarization in an earlier time period (0–10 min), the fluorescence in the cells was analyzed using a microplate fluorometer (Fluoroskan Ascent CF; Labsystems, Helsinki, Finland). The data were expressed as *F/F_0_*, where *F_0_* is the fluorescence in unstimulated cells and *F* is the fluorescence in stimulated cells.

### Determination of cell death by fluorescent microscopy

Cells (1×10^4^) were placed on 8-chamber coverslips (Asahi Glass Co., Tokyo, Japan) and treated with the agents to be tested for 24 h at 37°C in a 5% CO_2_-containing atmosphere. After removal of the medium, the cells were stained with 4 *μ*M each of calcein- AM and ethidium bromide homodimer-1 to label live and dead cells, respectively, using a commercially available kit (Live/Dead^®^ Viability/Cytotoxicity Kit; Invitrogen, Carlsbad, CA, USA) according to the manufacturer’s instructions. Images were obtained with a fluorescence microscope (IX71 inverted microscope, Olympus, Tokyo, Japan) and analyzed using the LuminaVision software (Mitani Corporation, Fukui, Japan).

### Determination of apoptotic cell death

Apoptotic cell death was quantitatively assessed by staining with propidium iodide (PI) and FITC-conjugated annexin V, as previously described (16). Briefly, cells plated in 24-well plates (2×10^5^ cells/well) were treated with TRAIL and the agents to be tested alone or together for specified times in DMEM containing 10% FBS (FBS/DMEM). Subsequently, the cells were stained with FITC-conjugated annexin V and PI using a commercially available kit (Annexin V FITC Apoptosis Detection Kit I; BD Biosciences) to label dead or damaged cells. The stained cells were evaluated in the FACSCalibur and analyzed using the CellQuest software. Four cellular subpopulations were evaluated: vital cells (annexin V^−^/PI^−^); early apoptotic cells (annexin V^+^/PI^−^); late apoptotic cells (annexin V^+^/PI^+^); and necrotic/damaged cells (annexin V^−^/PI^+^). Annexin V^+^ cells were considered to be apoptotic cells.

### Measurements of mitochondrial membrane potential (ΔΨ_m_) depolarization and caspase-3/7 activation

Caspase-3/7 activation and ΔΨ_m_ depolarization were simultaneously measured as previously described (16). Briefly, cells plated in 24-well plates (2×10^5^ cells/well) were treated with TRAIL and the agents to be tested alone or in combination in FBS/DMEM for 24 h. The cells were then stained with the dual sensor MitoCasp (Cell Technology Inc., Mountain View, CA). Caspase-3/7 activation and ΔΨm depolarization were determined using the FACSCalibur and the data were analyzed using the CellQuest software. In some experiments, changes in the ΔΨ_m_ were measured using the lipophilic cation JC-1 as previously described (17). Briefly, cells (5×10^5^ cells/500 *μ*l) were loaded with 2 *μ*M JC-1 at 37°C for 15 min, washed, and resuspended in HBSS. Following cell stimulation, the green fluorescence (monomeric JC-1) and red fluorescence (J-aggregates) were measured using the FL-1 and FL-2 channels, respectively, of the FACSCalibur.

### Measurement of caspase-12 activation

Activated caspase-12 in living cells was detected using the caspase-12 inhibitor ATAD-fmk conjugated to FITC as a marker, since this compound binds to active caspase-12, but not to inactive caspase-12. Cells (1×10^6^ cells/ml) were stained with FITCATAD-fmk for 30 min at 37°C using a kit (CaspGlow Fluorescein Caspase-12 Staining Kit; BioVision) according to the manufacturer’s protocol. Fluorescence was determined using the FL-1 channel of the FACSCalibur and analyzed using the CellQuest software.

### Western blot analysis

Western blot analysis was carried out as previously described (18) with minor modifications. Cells were treated with the agents to be tested for 24 h at 37°C, washed, and lysed with SDS-sample buffer. Whole cell lysates were subjected to SDS-PAGE and then transferred onto polyvinylidene difluoride membranes (Nippon Millipore, Tokyo, Japan). The membranes were blocked with BlockAce (Dainippon Sumitomo Pharma, Osaka, Japan) at room temperature for 60 min, washed, incubated with antibodies against GRP78, XBP-1, and caspase-12 overnight at 4°C and then incubated with horse-radish peroxidase-conjugated anti-rabbit IgG for 60 min at room temperature. After extensive washing, the immunoreactive proteins on the membranes were detected using the ECL Prime Western Blotting Reagent (GE Healthcare Japan, Tokyo, Japan). To verify equal loading, the membranes were reprobed with an anti-β-actin antibody. The signal intensities were quantified relative to the intensity of the β-actin signal using the NIH image software (NIH, Bethesda, MD).

### Statistical analysis

The statistical significance of differences among values was analyzed by one-way ANOVA followed by Tukey’s post hoc test. Values of P<0.05 were considered to indicate a statistically significant difference.

## Results

### TRAIL induces depolarization in human melanoma cells during apoptosis

**Figure 1:**
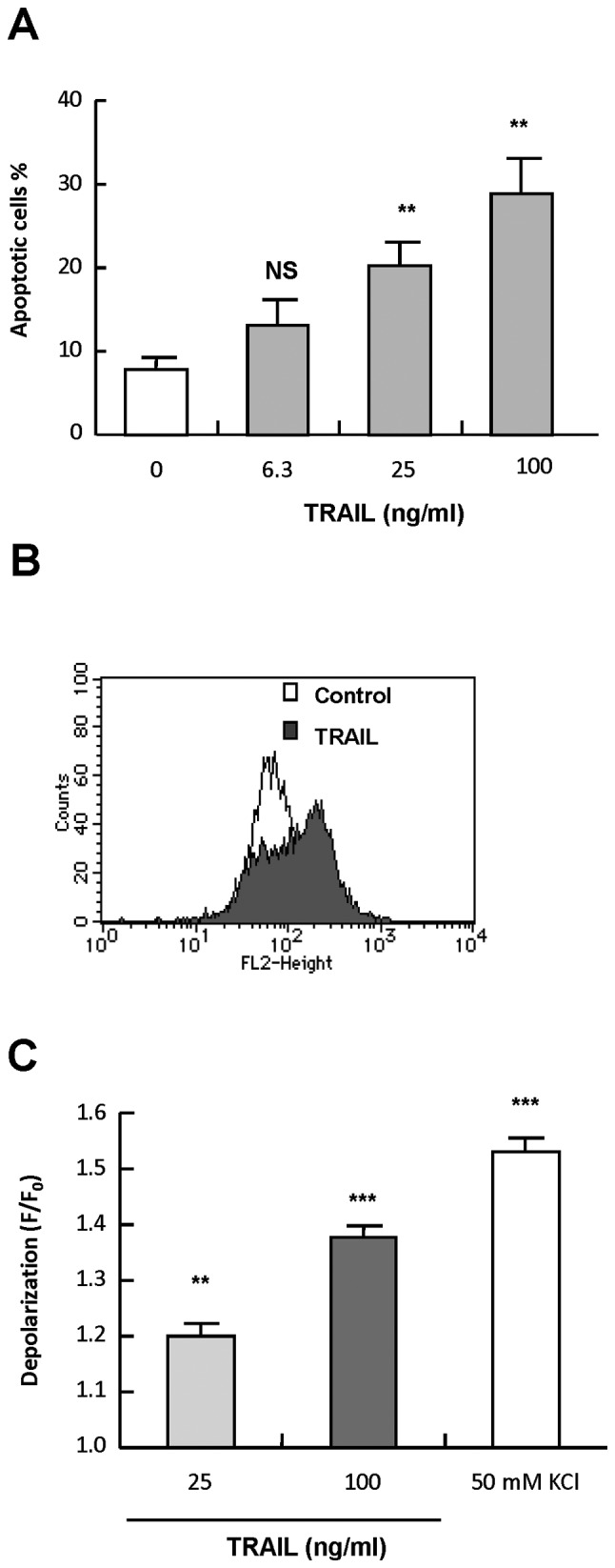
TRAIL induces membrane depolarization during apoptosis in human melanoma cells. (A) Human A375 melanoma cells were treated with TRAIL at the indicated concentrations for 24 h, stained with annexin V-FITC and PI, and analyzed by flow cytometry. Annexin V^+^ cells were considered to be apoptotic cells. The data represent the means ± SE from four independent experiments. ^**^P<0.01; NS, not significant. (B,C) A375 cells that had been loaded with *bis*-oxonol were treated with 25 and 100 ng/ml TRAIL for 4 h and analyzed for their fluorescence by flow cytometry. KCl was used as a positive control. A typical histogram observed in 100 ng/ml TRAIL-treated cells is shown in (B). The summarized data shown in (C) were expressed as *F/F_0_*, where *F_0_* is the fluorescence in unstimulated cells and *F* is the fluorescence in stimulated cells, and represent the means ± SE from four independent experiments. ^**^P<0.01; ^***^P<0.001.

Human melanoma cell line A375 cells were treated with varying concentrations of recombinant human TRAIL for 24 h, stained with annexin V-FITC and PI, and analyzed by flow cytometry. Treatment with TRAIL at concentrations of ≥25 ng/ml resulted in apoptotic (annexin V^+^) cells in a dose-dependent manner (Fig. 1A). Next, we examined whether TRAIL induced depolarization in the cells. The cells were treated with 25 and 100 ng/ml TRAIL for various times, and depolarization of the plasma membrane potential was measured by flow cytometry using the anionic dye *bis*-oxonol or DiBAC_4_(3). Essentially similar results were obtained with the two dyes. K^+^ loading (50 mM) resulted in significant (maximum of 2.2-fold) increase in the fluorescence, indicating the occurrence of depolarization. The fluorescence peaked within 5 min, and declined thereafter, but remained higher than the basal level up to 4 h (1.5-fold). Although TRAIL induced depolarization in a dose- and time-dependent manner, unlike K^+^ loading, it was observed after 2–4 h of treatment. At 4 h, 25 and 100 ng/ml TRAIL caused small but significant depolarization (1.2- and 1.4-fold, respectively) (Fig. 1B and C). TRAIL also induced robust depolarization within 4 h in SK-MEL-2 melanoma cells and Jurkat leukemia cells at concentrations ranging from 25–100 ng/ml (data not shown). These data show that TRAIL induces depolarization in human cancer cells during apoptosis. In the following experiments, we explored the role of depolarization in TRAIL-induced apoptosis using melanoma cells as model cell systems.

### K^+^ loading sensitizes melanoma cells to TRAIL-induced apoptosis

**Figure 2:**
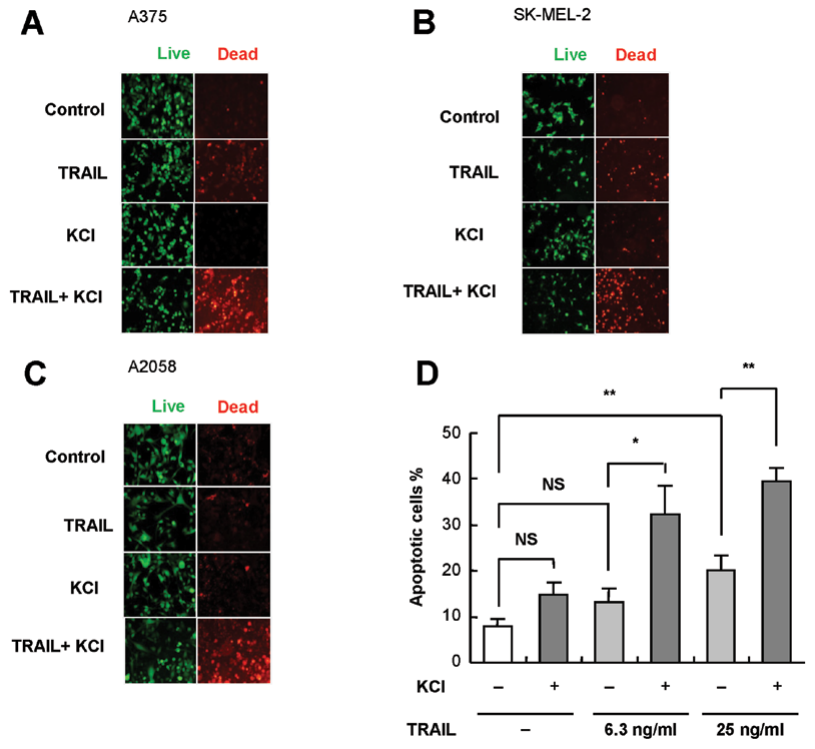
K^+^ loading sensitizes human melanoma cells to TRAIL-induced apoptosis. (A–C) A375 (A), SK-MEL-2 (B) and A2058 (C) cells were treated with 25 ng/ml TRAIL and 50 mM KCl alone or in combination for 24 h. After removal of the medium, the cells were stained with calcein-AM and ethidium bromide homodimer to label live cells (green) and dead cells with compromised cell membranes (red), respectively. Images were obtained with a fluorescence microscope (×100). The results shown are representative of four independent experiments. (D) After treatment with 6.3 and 25 ng/ml TRAIL and KCl alone or in combination for 24 h, A375 cells were stained with annexin V-FITC and PI and analyzed by flow cytometry. The data represent means ± SE from four independent experiments. ^*^P<0.05; ^**^P<0.01; NS, not significant.

Next, we examined whether the modulation of depolarization affected TRAIL cytotoxicity. To this end, persistent depolarization was induced by extracellular high K^+^ loading. Following treatment with TRAIL and K^+^ alone or in combination for 24 h, the cells were stained with calcein-AM and ethidium bromide homodimer, and observed under a fluorescence microscopy. Live cells were stained green with calcein-AM, whereas dead cells with compromised cell membranes were stained red with ethidium bromide homodimer. TRAIL and K^+^ alone had minimal or weak cytotoxicity. However, the combined use of the two agents resulted in considerable cell death (Fig. 2A). Similar synergistic effects were observed in other TRAIL-sensitive SK-MEL-2 cells as well as in TRAIL-resistant A2058 cells (Fig. 2B and C). As shown in Fig. 2D, K^+^ alone caused minimal apoptosis but significantly enhanced TRAIL-induced apoptosis. These data show that K^+^ loading sensitizes melanoma cells to TRAIL-induced apoptosis.

### K_ATP_ channel inhibitors specifically sensitize melanoma cells to TRAIL-induced apoptosis

**Figure 3:**
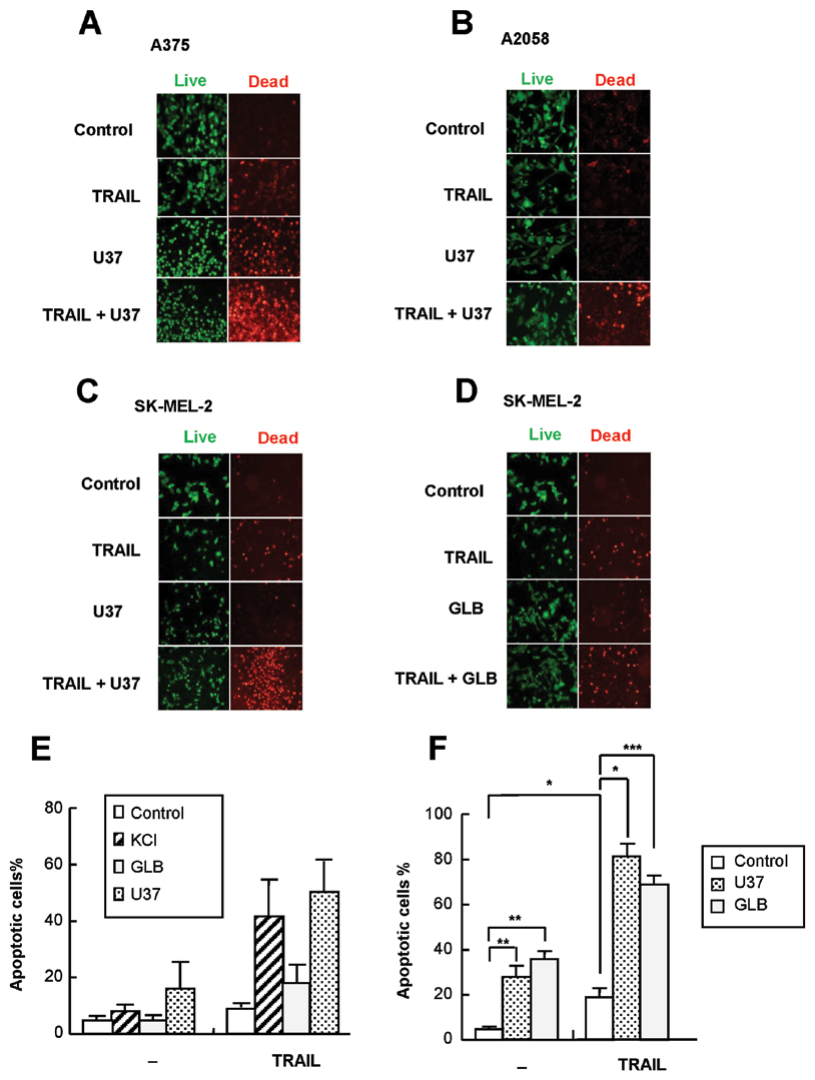
K_ATP_ channel inhibitors sensitize human melanoma cells to TRAIL-induced apoptosis. (A–D) A375 (A), A2058 (B) and SK-MEL-2 (C,D) cells were treated with TRAIL and U37883A (U37) or glibenclamide (GLB) alone or in combination for 24 h, stained with calcein-AM and ethidium bromide homodimer and observed under a fluorescence microscope (×100). The results shown are representative of three independent experiments. (E,F) After treatment of A375 cells with TRAIL and U37 or GLB alone or in combination for 24 h (E) or 72 h (F), apoptotic cell death was evaluated by flow cytometry using annexin V-FITC and PI staining. The data represent the means ± SE from four independent experiments. ^*^P<0.05; ^**^P<0.01; ^***^P<0.001.

**Figure 4:**
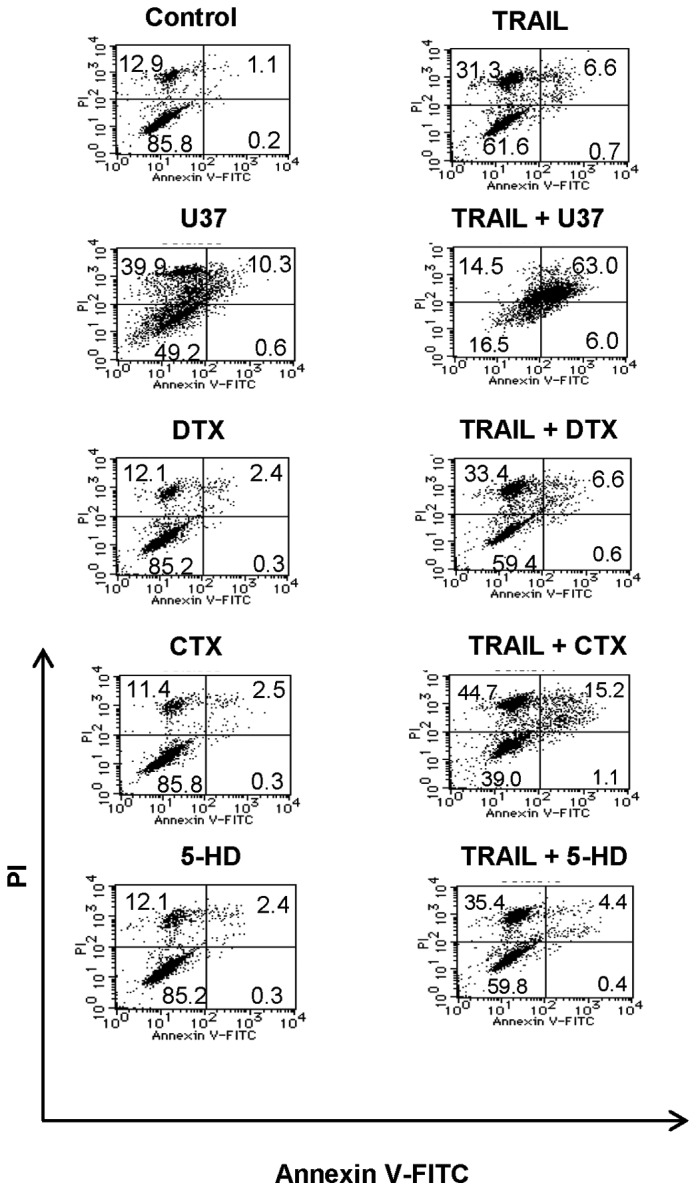
Inhibitors of other K^+^ channels have minimal effect on TRAIL-induced apoptosis. A375 cells were treated with TRAIL and U37883A (U37), α-dendrotoxin (DTX), charybdotoxin (CTX) and 5-hydroxydecanoate (5-HD) alone or in combination for 24 h, and apoptotic cell death was measured by flow cytometry using annexin V-FITC and PI staining. The data are representative of three independent experiments.

Since K^+^ efflux through K^+^ channels results in repolarization, blockade of the K^+^ efflux is necessary for persistent depolarization. Such a blockade can be pharmacologically achieved by inhibition of channels such as K_ATP_(9). Therefore, we next examined the effect of K_ATP_ channel-specific inhibitors U37 and GLB on TRAIL cytotoxicity by fluorescence microscopy. After 24 h of treatment, U37 alone showed substantial cytotoxicity toward several melanoma cell lines and significantly enhanced TRAIL cytotoxicity toward all cell lines tested, including A375, A2058 and SK-MEL-2 (Fig. 3A–C). On the contrary, GLB had a marginal effect on TRAIL cytotoxicity in SK-MEL-2 cells (Fig. 3D) and A375 cells (data not shown). In agreement with these observations, U37, but not GLB, caused TRAIL-induced apoptosis at that time (Fig. 3E). On the other hand, after 72 h of treatment, U37 and GLB alone enhanced robust apoptosis. Necrotic/damaged (annexin V^−^/PI^+^) cells often increased significantly. In addition, both agents significantly enhanced TRAIL-induced apoptosis (Fig. 3F). To determine whether such effect was specific for K_ATP_ channel inhibitors, inhibitors of other types of K^+^ channels were examined for their effects on TRAIL-induced apoptosis. The K_v_-specific inhibitor DTX and K_Ca_-specific inhibitor CTX had minimal effects on the apoptosis up to 72 h (Fig. 4). The mitochondrial K_ATP_ inhibitor 5-HD had no effect either (Fig. 4), suggesting that plasma membrane K_ATP_ is specifically involved in the sensitization. Moreover, TEA, which mainly inhibits K_v_ and K_Ca_, was also ineffective (data not shown). U37 was as effective as 100 ng/ml TRAIL in causing depolarization, whereas GLB had a smaller effect (maximum of 1.3-fold). On the other hand, TEA caused no significant depolarization. Taken together, these data show that K_ATP_ channel inhibitors specifically sensitize melanoma cells to TRAIL-induced apoptosis.

### Melanocytes are insensitive to TRAIL-induced depolarization and apoptosis and to sensitization by membrane-depolarizing agents

**Figure 5:**
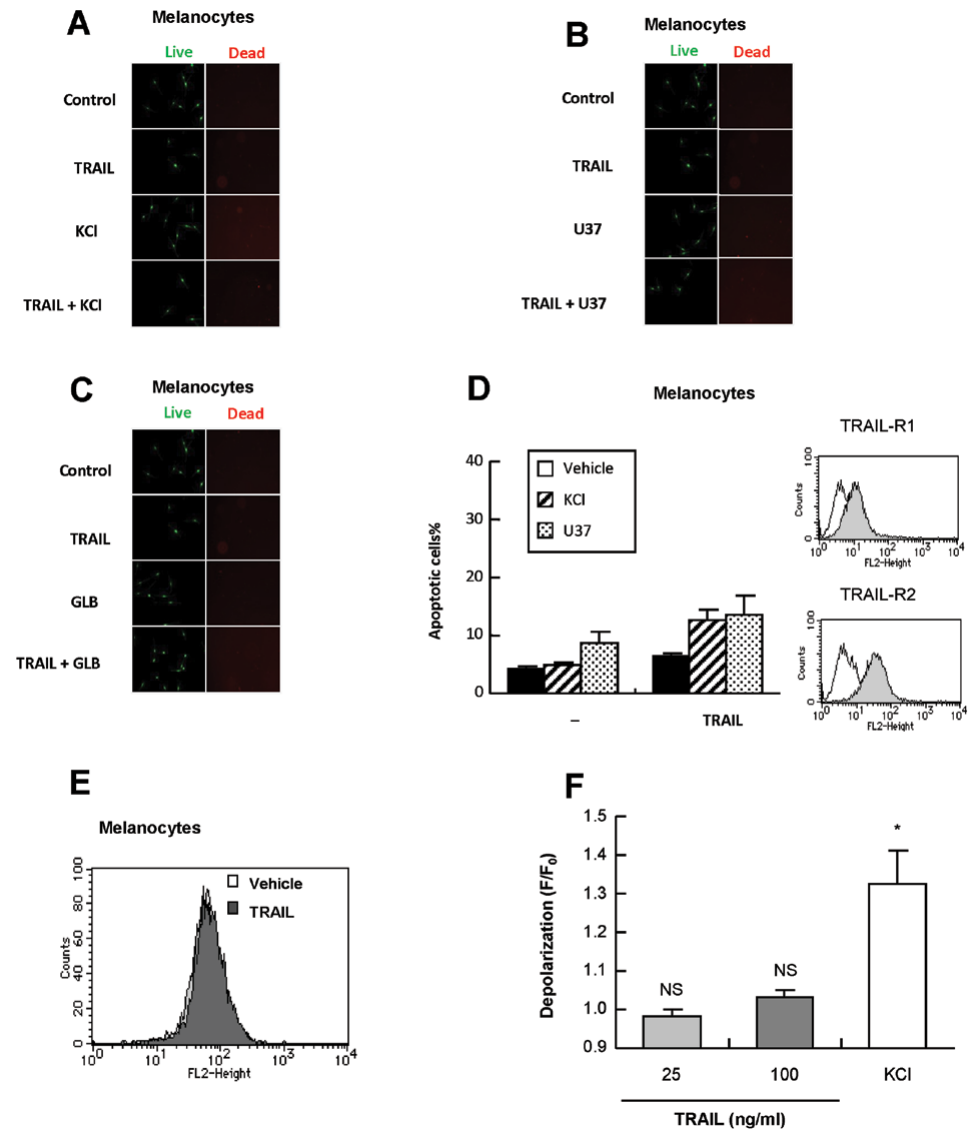
Melanocytes are insensitive to TRAIL-induced depolarization and apoptosis and sensitization by membrane-depolarizing agents. (A–C) Melanocytes were treated with TRAIL and KCl, U37883A (U37) or glibenclamide (GLB) alone or in combination for 24 h, stained with calcein-AM and ethidium bromide homodimer and observed under a fluorescence microscope (×100). (D) (Left panel) The cells treated as described above were analyzed for their apoptotic cell death. The results shown are representative of four independent experiments. (Right panel) Intact melanocytes were analyzed for their expression of TRAIL-R1 and TRAIL-R2 by flow cytometry using indirect immunofluorescence. (E,F) Melanocytes were treated with 25 and 100 ng/ml TRAIL or KCl for 4 h and depolarization was measured by flow cytometry using *bis*-oxonol. A typical histogram is shown in (E). The summarized data shown in (F) were expressed as *F/F_0_*, where *F_0_* is the fluorescence in unstimulated cells and *F* is the fluorescence in stimulated cells, and represent the means ± SE from four independent experiments.^*^P<0.05

TRAIL has been shown to induce apoptosis in cancer cells with minimal cytotoxicity in non-transformed cells. Therefore, we determined whether the membrane-depolarizing agents affected the tumor-selectivity. Treatment with melanocytes with TRAIL, K^+^, U37 and GLB alone or in combination resulted in minimal apoptosis and cytotoxicity (Fig. 5A–D), although they expressed substantial levels of DR4 and DR5 on the cell surface (Fig. 5D). TRAIL induced minimal depolarization in melanocytes (Fig. 5E and F). On the other hand, K^+^ loading resulted in substantial depolarization in the cells, which was comparable to that observed in A375 cells. Taken together, these data show that despite their substantial expressions of DRs, melanocytes are insensitive to TRAIL-induced depolarization and apoptosis as well as to sensitization by the membrane-depolarizing agents.

### The amplification of apoptosis is associated with increased ΔΨ_m_ collapse and caspase-3/7 activation

**Figure 6:**
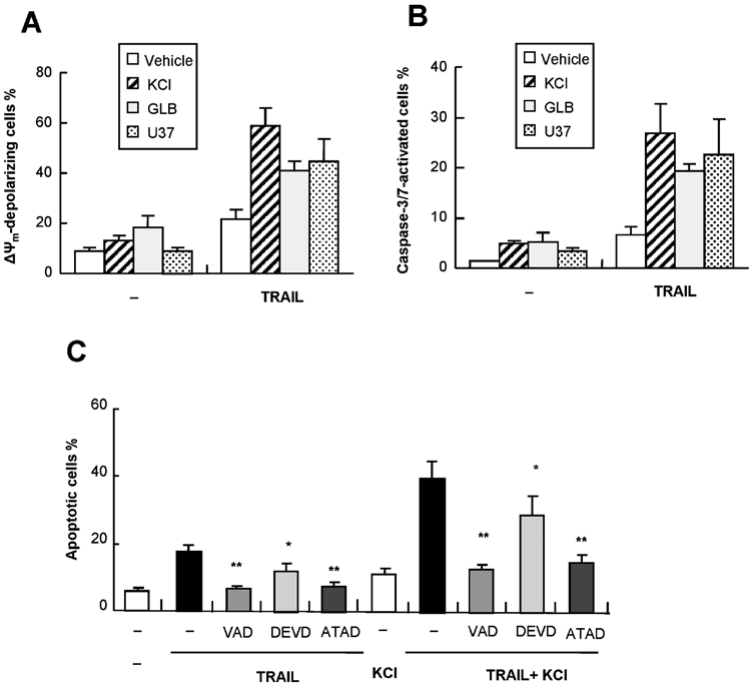
The intrinsic apoptotic pathway is involved in, but is not sufficient for, the sensitization to TRAIL-induced apoptosis in melanoma cells. (A,B) A375 cells were treated with TRAIL and U37883A (U37), glibenclamide (GLB) or KCl alone or in combination for 24 h. Caspase-3/7 activation and ΔΨ_m_ depolarization were determined by flow cytometry. The data are representative of three independent experiments. (C) A375 cells were treated with 25 ng/ml TRAIL and KCl alone or in combination in the presence or absence of z-VAD-fmk (VAD), z-DEVD-fmk (DEVD) or z-ATAD-fmk (ATAD) for 24 h and apoptotic cells were evaluated by flow cytometry using annexin V-FITC and PI staining. The data represent the means ± SE from five independent experiments. ^*^P<0.05; ^**^P<0.01.

Flow cytometric analysis using specific fluorescent probes revealed that TRAIL-induced apoptosis was accompanied by a loss of ΔΨ_m_ and activation of caspase-3/7, both of which were augmented by K^+^, U37, and GLB (Fig. 6A and B). The ΔΨ_m_ depolarization was confirmed by another probe JC-1 (data not shown). The data showed that despite its weak effect on TRAIL-induced apoptosis during the initial 24 h, GLB can enhance the ΔΨ_m_ collapse and caspase-3/7 activation, suggesting that the intrinsic apoptotic pathway is involved in, but is not sufficient for, the amplification of apoptosis. To explore the role of caspase-3/7 further, we examined the effect of the caspase-3/7-specific inhibitor z-DEVD-fmk on apoptosis. As shown in Fig. 6C, the general caspase inhibitor VAD completely blocked TRAIL-induced apoptosis. The caspase-12-specific inhibitor ATAD exhibited similar effects, whereas DEVD inhibited the apoptosis marginally. On the other hand, ATAD and to a lesser extent DEVD reduced the K^+^-mediated amplification of apoptosis (Fig. 6C), suggesting that caspase-12 as well as caspase-3 play a role in the amplification of apoptosis.

### Role of ER stress and caspase-12 in the amplification of apoptosis

**Figure 7:**
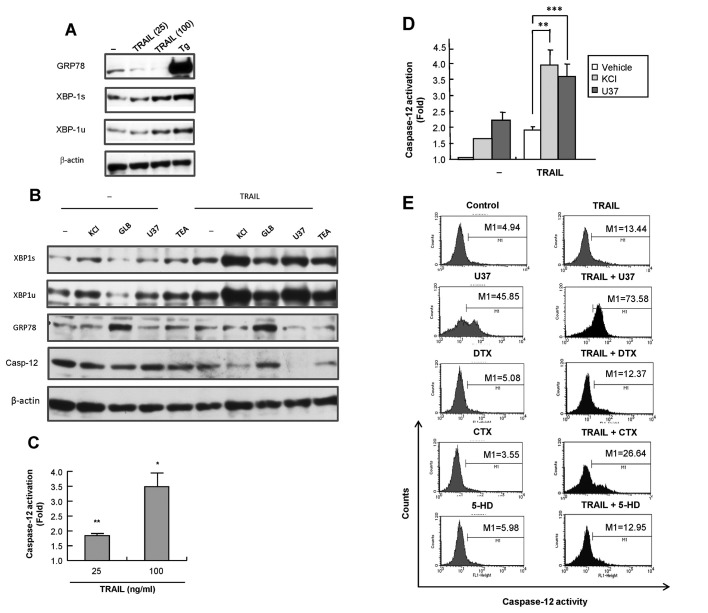
TRAIL induces ER stress and caspase-12 activation and the membrane-depolarizing agents potentiate these effects. (A) A375 cells were treated with 25 and 100 ng/ml TRAIL or 1 *μ*M Tg for 24 h. (B) A375 cells were treated with TRAIL and KCl, U37883A (U37), glibenclamide (GLB) and TEA alone or in combination for 24 h. The cells were then washed, lysed with SDS-sample buffer and analyzed for their contents of GRP78, XBP-1 and/or full-length caspase-12 by western blot analysis with specific antibodies. To verify equal loading, the blots were re-probed with an anti-β-actin antibody. The data are representative of three independent experiments. (C,D) A375 cells were treated with TRAIL (C) or TRAIL, KCl and U37883A (U37) alone or in combination for 24 h and caspase-12 activity was measured using FITC-ATAD-fmk. The data are shown as ratios to the basal activity and represent the means ± SE from three independent experiments. ^*^P<0.05; ^**^P<0.01; ^***^P<0.001. (E) A375 cells were treated with TRAIL and U37883A (U37), α-dendrotoxin (DTX), charybdotoxin (CTX) and 5-hydroxydecanoate (5-HD) alone or in combination for 72 h, and caspase-12 activity was measured by flow cytometry using FITC-ATAD-fmk. The data are representative of two independent experiments.

Caspase-12 is ubiquitously expressed and localized to the ER membrane, and is specifically activated by ER stress to play a key role in stress-induced apoptosis (23). Therefore, we hypothesized that the ER stress-mediated apoptotic pathway, including caspase-12, was involved in the amplification of TRAIL-induced apoptosis. To test this hypothesis, we analyzed the unfolded protein response (UPR), a cellular response to ER stress. A375 cells were treated with 25 and 100 ng/ml TRAIL for 24 h and their expression of two UPR proteins, GRP78 and XBP1 were analysed by western blotting. Tg, an inhibitor of sarco/endoplasmic reticulum Ca^2+^-ATPase, was shown to be a potent inducer of GRP78 expression in human melanoma cells (22) and served as a positive control. Tg treatment caused a robust increase in the expression of GRP78, while TRAIL treatment had a minimal effect on or often decreased this expression (Fig. 7A). On the other hand, TRAIL dose-dependently increased the expression of both the inactive unspliced form of XBP-1 (XBP-1u) and the active spliced form of XBP-1 (XBP-1s) (maximum of 1.8- and 2.1-fold, respectively), indicating the activation of XBP-1. Then, we analyzed the effect of membrane-depolarizing agents on TRAIL-induced UPR response. K^+^ and U37 synergized with TRAIL to increase the expression of both forms of XBP-1, whereas GLB and TEA had minimal effects (Fig. 7B). Next, we examined whether TRAIL induced activation of caspase-12 and whether membrane-depolarizing agents impacted the effect. The activation of caspase-12 was initially evaluated by detecting its processing by western blotting. As shown in Fig. 7B, K^+^ and U37, but not GLB or TEA, synergistically induced the processing of caspase-12, a hallmark of its activation. The functional activation of caspase-12 was assessed by measuring the conversion of a cell-permeable substrate, FITC-ATAD-fmk. TRAIL induced the activation of caspase-12 in a dose-dependent manner at concentrations that induced apoptosis (Fig. 7C). In addition, K^+^ and U37 significantly enhanced the effect (Fig. 7D), whereas other K^+^ channel inhibitors had minimal effects even after 72 h of treatment (Fig. 7E). Taken together, these data show that the ER stress-mediated death pathway involving caspase-12 plays an important role in the amplification of TRAIL-induced apoptosis.

## Discussion

The data presented in this paper show that TRAIL induces robust depolarization of the plasma membrane potential in human melanoma cells and that membrane-depolarizing agents such as K^+^ and K_ATP_ channel-selective inhibitors potentiate TRAIL-induced apoptosis. K_ATP_ channels by themselves caused apoptosis under a long-time treatment, suggesting that persistent depolarization alone trigger apoptosis. K_ATP_ channels appeared to play a specific role in the regulation of apoptosis, since inhibitors of other types of K^+^ channels, including mitochondrial K_ATP_, K_v_ and K_Ca_ channels exhibited no effects on apoptosis. Of note, TRAIL caused minimal membrane depolarization and apoptosis in melanocytes despite their substantial expression of DR4 and DR5, and the cells showed unresponsiveness to K_ATP_ channel inhibitors. K^+^ caused robust depolarization in melanocytes, indicating depolarization machineries are intact. Thus, our data show that human melanoma cells are far more susceptible than melanocytes to the K_ATP_ channel-regulated apoptotic pathway.

The intrinsic mitochondrial pathway plays a crucial role in amplifying TRAIL-induced apoptosis, and collapse of the ΔΨ_m_ is considered to be a hallmark of this pathway, although it is still a matter of debate whether this event is a cause or a result of permeabilization of the outer mitochondrial membrane (23–25). TRAIL induced both the ΔΨ_m_ collapse and the caspase-3/7 activation in melanoma cells, and the sensitization of TRAIL-induced apoptosis was associated with their enhancement, indicating the involvement of the intrinsic pathway. However, this pathway appeared not to be sufficient for the sensitization, due to the fact that GLB upregulated both events but had minimal effects on TRAIL-induced apoptosis during the initial 24 h. On the other hand, K^+^ and U37 rapidly sensitized melanoma cells to TRAIL-induced apoptosis within 24 h. Therefore, it is possible to speculate that another amplification pathway, which is activated by K^+^ and U37, but not GLB, may be required for the rapid sensitization. Consistent with this view, we found that TRAIL-induced apoptosis involved activation of caspase-12, which was enhanced by K^+^ and U37, but not GLB. Caspase-12 has been shown to be predominantly located at the outer membrane of the ER, to be specifically activated by ER stress-inducing agents and to play an important role in the apoptosis induced by ER stress (19,20,26,27). The emerging view is that, besides mitochondria, the ER is another key player in the regulation of apoptosis induced by a variety of death stimuli, including TRAIL. Disparate perturbations in its normal functions, such as the accumulation of unfolded or misfolded proteins, ER lipid imbalances or changes in the redox balance or Ca^2+^ conditions in the ER lumen, trigger ER stress (20,26–28). The cells then activate ER stress responses, including the UPR, to alleviate the stress, but an excessive and prolonged UPR leads to apoptosis (27–29). The UPR involves transcription-dependent upregulation of ER-resident chaperones, and the ER chaperone GRP78 plays a central role in this response. Upon ER stress, GRP78 dissociates from ER transmembrane proteins, such as inositol requiring 1 and activating transcription factor 6, to bind to unfolded or misfolded proteins, resulting in aggregation of the transmembrane proteins and their activation. Activated inositol requiring 1 splices the mRNA for XBP-1 to allow translation of the mature XBP-1 protein, which acts as a transcription factor and mediates the transcriptional upregulation of numerous genes involved in ER function (30). Consistent with the role of ER stress in TRAIL-induced apoptosis and the sensitization, we found that TRAIL and U37 induced activation of XBP-1. Collectively, our data strongly suggest that ER stress-induced activation of caspase-12 is involved in TRAIL-induced apoptosis and sensitization. The human caspase-12 homologue exists on chromosome 11 and is 68% homology with murine caspase-12 and shows 57% homology with human caspase-4 (31). Owing to multiple mutations, this gene does not seem to be expressed in a functional form in humans, and therefore the role of caspase-12 in the induction of apoptosis in human cells is controversial (27,31). However, there are multiple possibilities that human cells express caspase-12-like activity, such as (i) a different locus in the human genome carries a functional caspase-12 gene, (ii) its function is carried out by other caspases; and (iii) mRNA editing or other mechanisms can override frame-shift mutations (32). Hence further studies are needed to clarify the role of caspase-12 in ER stress-induced apoptosis. In support of this view, there is an increasing body of evidence suggesting that a caspase-12-like protein exists and is activated in human cells following the induction of ER stress by divergent causes, including cisplatin, tetrocarcin A and hyperthermia (32–36). In the present study, we have demonstrated the existence of full-length caspase-12 in human melanoma cells and its processing coincided with the activation of caspase-12 activity. These findings provide further evidence for a role of caspase-12 in ER stress-induced apoptosis. It should be noted that caspase-12 is directly activated by the Ca^2+^-dependent protease calpain. Upon apoptotic stimulation, calpain cleaves the regulatory prodomain, thereby activating caspase-12, and the activated caspase-12 directly cleaves caspase-9 for its activation without the need for release of cytochrome c and apoptotic protease-activating factor-1, which in turn activates caspase-3 (26,37,38). Accordingly, caspase-12 can be activated before caspase-9 and caspase-3/7.

Similar to caspase-12 in rodents, caspase-4 in humans has been shown to be predominantly located on the outer membrane of the ER and to play an important role in ER stress-induced apoptosis (20,30). Caspase-4 was recently shown to be involved in TRAIL-induced apoptosis of human melanoma cells. However, inhibition of caspase-4 partially, but not completely, blocked apoptosis (39), suggesting that its role is limited. Since caspase-4 has been shown to be activated later than caspase-8, caspase-9 and caspase-3 (39), it is possible that caspase-4 and caspase-12 may play roles in different processes/stages in ER stress-induced apoptosis. Our preliminary attempt to establish caspase-12 gene knockdown cells was hampered by considerable cell death in the resting state. On the other hand, the caspase-12 inhibitor had a minimal effect on their survival. These observations suggest that caspase-12 may have an important function in human melanoma survival that is independent of its enzyme activity. Further studies are needed to fully elucidate the role of caspase-12 in the TRAIL-induced apoptosis of human tumor cells.

In conclusion, we have demonstrated for the first time the pro-apoptotic role of depolarization in TRAIL-induced apoptosis via ER stress. Since melanoma cells are far more susceptible to the depolarization-mediated apoptosis than melanocytes, membrane-depolarizing drugs such as K_ATP_ channel inhibitors may exhibit cytotoxicity in a tumor-selective manner and have therapeutic potential in the treatment of TRAIL-resistant melanoma cells.

## References

[1] LeBlanc HN, Ashkenazi A (2003). Apo2L/TRAIL and its death and decoy receptors. Cell Death Differ.

[2] Kischkel FC, Lawrence DA, Chuntharapai A (2000). Apo2L/TRAIL-dependent recruitment of endogenous FADD and caspase-8 to death receptors 4 and 5. Immunity.

[3] Lavrik IN, Golks A, Krammer PH (2005). Caspases: pharmacological manipulation of cell death. J Clin Invest.

[4] Danial NN, Korsmeyer SJ (2004). Cell death: critical control points. Cell.

[5] Yan N, Shi Y (2005). Mechanisms of apoptosis through structural biology. Annu Rev Cell Dev Biol.

[6] Dyer MJ, MacFarlane M (2007). Cohen GM. Barriers to effective TRAIL-targeted therapy of malignancy. J Clin Oncol.

[7] McCarthy JV, Cotter TG (1997). Cell shrinkage and apoptosis: a role for potassium and sodium ion efflux. Cell Death Differ.

[8] Lang F, Föller M, Lang K (2007). Cell volume regulatory ion channels in cell proliferation and cell death. Methods Enzymol.

[9] Burg ED, Remillard CV, Yuan JX (2008). Potassium channels in the regulation of pulmonary artery smooth muscle cell proliferation and apoptosis: pharmacotherapeutic implications. Br J Pharmacol.

[10] Bortner CD, Gomez-Angelats M, Cidlowski JA (2001). Plasma membrane depolarization without repolarization is an early molecular event in anti-Fas-induced apoptosis. J Biol Chem.

[11] Yin W, Li X, Feng S (2009). Plasma membrane depolarization and Na, K-ATPase impairment induced by mitochondrial toxins augment leukemia cell apoptosis via a novel mitochondrial amplification mechanism. Biochem Pharmacol.

[12] Nolte F, Friedrich O, Rojewski M, Fink RH, Schrezenmeier H, Körper S (2004). Depolarisation of the plasma membrane in the arsenic trioxide (As_2_O_3_)-and anti-CD95-induced apoptosis in myeloid cells. FEBS Lett.

[13] Ghoumari AM, Piochon C, Tomkiewicz C (2006). Neuroprotective effect of mifepristone involves neuron depolarization. FASEB J.

[14] Karki P, Seong C, Kim JE (2007). Intracellular K^+^ inhibits apoptosis by suppressing the Apaf-1 apoptosome formation and subsequent downstream pathways but not cytochrome c release. Cell Death Differ.

[15] Yoshimaru T, Suzuki Y, Inoue T, Ra C (2009). L-type Ca^2+^ channels in mast cells: activation by membrane depolarization and distinct roles in regulating mediator release from store-operated Ca^2+^ channels. Mol Immunol.

[16] Suzuki Y, Yoshimaru T, Inoue T, Ra C (2009). Cav 1.2 L-type Ca^2+^ channel protects mast cells against activation-induced cell death by preventing mitochondrial integrity disruption. Mol Immunol.

[17] Suzuki Y, Yoshimaru T, Inoue T, Ra C (2006). Mitochondrial Ca^2+^ flux is a critical determinant of the Ca^2+^ dependence of mast cell degranulation. J Leukoc Biol.

[18] Inoue T, Suzuki Y, Yoshimaru T, Ra C (2008). Nitric oxide protects mast cells from activation-induced cell death: the role of the phosphatidylinositol-3 kinase-Akt-endothelial nitric oxide synthase pathway. J Leukoc Biol.

[19] Nakagawa T, Zhu H, Morishima N, Li E, Xu J, Yankner BA, Yuan J (2000). Caspase-12 mediates endoplasmic-reticulum-specific apoptosis and cytotoxicity by amyloid beta. Nature.

[20] Boyce M, Yuan J (2000). Cellular response to endoplasmic reticulum stress: a matter of life or death. Cell Death Differ.

[21] Breckenridge DG, Germain M, Mathai JP, Nguyen M, Shore GC (2003). Regulation of apoptosis by endoplasmic reticulum pathways. Oncogene.

[22] Chen LH, Jiang CC, Kiejda KA (2007). Thapsigargin sensitizes human melanoma cells to TRAIL-induced apoptosis by up-regulation of TRAIL-R2 through the unfolded protein response. Carcinogenesis.

[23] Cai J, Yang J, Jones DP (1998). Mitochondrial control of apoptosis: the role of cytochrome c. Biochim Biophys Acta.

[24] Düssmann H, Rehm M, Kögel D, Prehn JH (2003). Outer mitochondrial membrane permeabilization during apoptosis triggers caspase-independent mitochondrial and caspase-dependent plasma membrane potential depolarization: a single-cell analysis. J Cell Sci.

[25] Adachi S, Cross AR, Babior BM, Gottlieb RA (1997). Bcl-2 and the outer mitochondrial membrane in the inactivation of cytochrome c during Fas-mediated apoptosis. J Biol Chem.

[26] Groenendyk J, Michalak M (2005). Endoplasmic reticulum quality control and apoptosis. Acta Biochim Pol.

[27] Szegezdi E, Fitzgerald U, Samali A (2003). Caspase-12 and ER-stress-mediated apoptosis: the story so far. Ann NY Acad Sci.

[28] Rutkowski DT, Kaufman RJ (2004). A trip to the ER: coping with stress. Trends Cell Biol.

[29] Ferri KF, Kroemer G (2001). Organelle-specific initiation of cell death pathways. Nat Cell Biol.

[30] Jiang CC, Chen LH, Gillespie S (2007). Tunicamycin sensitizes human melanoma cells to tumor necrosis factor-related apoptosis-inducing ligand-induced apoptosis by up-regulation of TRAIL-R2 via the unfolded protein response. Cancer Res.

[31] Fischer H, Koenig U, Eckhart L, Tschachler E (2002). Human caspase 12 has acquired deleterious mutations. Biochem Biophys Res Commun.

[32] Mandic A, Hansson J, Linder S, Shoshan MC (2003). Cisplatin induces endoplasmic reticulum stress and nucleus-independent apoptotic signaling. J Biol Chem.

[33] Tinhofer I, Anether G, Senfter M (2002). Stressful death of T-ALL tumor cells after treatment with the anti-tumor agent Tetrocarcin-A. FASEB J.

[34] Xie Q, Khaoustov VI, Chung CC (2002). Effect of tauroursodeoxycholic acid on endoplasmic reticulum stress-induced caspase-12 activation. Hepatology.

[35] Trisciuoglio D, Uranchimeg B, Cardellina JH (2008). Induction of apoptosis in human cancer cells by candidaspongiolide, a novel sponge polyketide. J Natl Cancer Inst.

[36] Shellman Y, Howe WR, Miller LA (2007). Hyperthermia induces endoplasmic reticulum-mediated apoptosis in melanoma and non-melanoma skin cancer cells. J Invest Dermatol.

[37] Rao RV, Castro-Obregon S, Frankowski H (2002). Coupling endoplasmic reticulum stress to the cell death program. An Apaf-1-independent intrinsic pathway. J Biol Chem.

[38] Morishima N, Nakanishi K, Takenouchi H, Shibata T, Yasuhiko Y (2002). An endoplasmic reticulum stress-specific caspase cascade in apoptosis. Cytochrome c-independent activation of caspase-9 by caspase-12. J Biol Chem.

[39] Mao ZG, Jiang CC, Yang F, Thorne RF, Hersey P, Zhang XD (2010). TRAIL-induced apoptosis of human melanoma cells involves activation of caspase-4. Apoptosis.

